# Eag1 Channels as Potential Cancer Biomarkers

**DOI:** 10.3390/s120505986

**Published:** 2012-05-10

**Authors:** Jesús Adrián Rodríguez-Rasgado, Isabel Acuña-Macías, Javier Camacho

**Affiliations:** Department of Pharmacology, Centro de Investigación y Estudios Avanzados del IPN, Avenida Instituto Politécnico Nacional 2508, Mexico City 07360, Mexico; E-Mails: jarodrigu@cinvestav.mx (J.A.R.-R.); isa_ratona@hotmail.com (I.A.-M.)

**Keywords:** *ether à-go-go*, tumor markers, prognostic markers, cervical cancer, human papilloma virus, estrogens, leukemia, colon cancer, potassium channels

## Abstract

Cancer is a leading cause of death worldwide. New early tumor markers are needed to treat the disease at curable stages. In addition, new therapeutic targets are required to treat patients not responding to available treatments. Ion channels play major roles in health and disease, including cancer. Actually, several ion channels have been suggested as potential tumor markers and therapeutic targets for different types of malignancies. One of most studied ion channels in cancer is the voltage-gated potassium channel Eag1 (*ether à go-go 1*), which has a high potential to be used as a cancer biomarker. Eag1 is expressed in most human tumors, in contrast to its restricted distribution in healthy tissues. Several findings suggest Eag1 as a potential early marker for cervical, colon, and breast cancer. In addition, because Eag1 amplification/expression is associated with poor survival in leukemia, colon and ovarian cancer patients, it has also been proposed as a prognosis marker. Moreover, inhibition of either expression or activity of Eag1 leads to reduced proliferation of cancer cells, making Eag1 a potential anticancer target. Using Eag1 in cancer detection programs could help to reduce mortality from this disease.

## 1. Introduction

**Figure 1.**
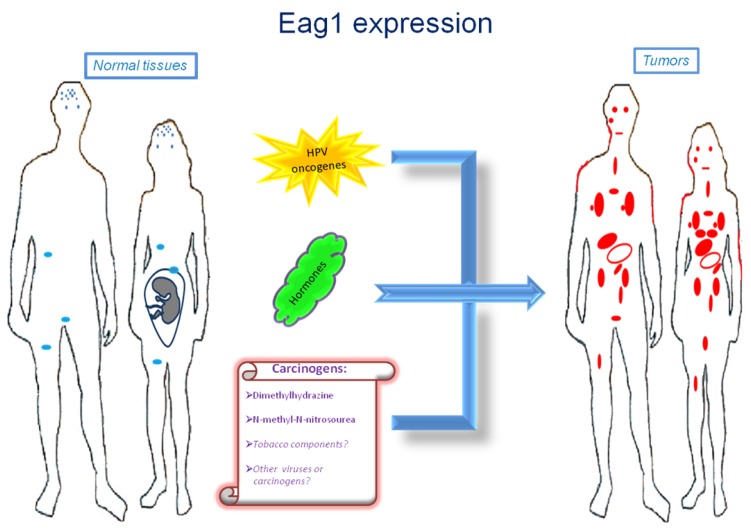
Eag1 channels as potential markers in oncology. Eag1 displays a restricted distribution in normal tissues but is more abundant in most tumors. The regulation of Eag1 channel expression by cancer-associated factors (*i.e.*, HPV oncogenes, hormones, and carcinogens) and the detection of Eag1 in pre-malignant lesions suggest this channel as a potential early tumor marker.

Ion channels are membrane proteins allowing the passage of ions. These proteins play major roles in human physiology including neural transmission, heart rate, muscle contraction, insulin secretion, immune function, and cell proliferation. The relevance of these proteins is highlighted when either their expression or activity is altered in different diseases, including cardiac arrhythmias, epilepsy, cystic fibrosis, hyperinsulinemia, deafness, and migraine [1]. Accordingly, ion channels are targets of a huge number of drugs used to treat several diseases. Specifically, voltage-gated potassium channels have been associated with several disorders and represent very interesting targets for many diseases including cancer [2]. Among these channels, *Ether à go-go-1* (Eag1) has gained great interest in cancer because of its restricted distribution in normal tissues, its role in tumor cell proliferation, its regulation by cancer etiological factors, and its association with poor survival. Excellent reviews on Eag1 channel properties and its association with cancer can be found in the literature [3–8]. Here we will focus mainly on the findings suggesting Eag1 channels as: (a) cancer markers, (b) potential early tumor markers, and (c) prognostic markers of the disease. Regulation of Eag1 channels by carcinogens and other cancer etiological factors, as well as their expression in cancer tissues (Figure 1) and their association with poor survival, make these proteins excellent oncology biomarkers both for diagnosis and therapy.

## 2. Eag1

Eag1 (Kv10.1, KCNH1) is a voltage-gated potassium channel whose locus was first described in a chromosomal screening in *Drosophila melanogaster* as being responsible for the leg rhythmic phenotype induced upon exposure to ether anesthesia [9–11]. The Eag1 channel has been cloned from several species, including rat, bovine and humans [12–16]. Eag1 distribution in normal tissues is very restricted. It is mainly expressed in the brain, but it can also be found in myoblasts, placenta, testes, and adrenal gland [14,16–18]. In myoblasts, Eag1 channel activity provides the hyperpolarization needed just before cell fusion [14]. Nevertheless, the precise role of Eag1 in most of the normal tissues where it is expressed remains unknown. The members of the Eag1 family share a very similar structure to that of other voltage-gated potassium channels. Eag1 channels have four identical α-subunits, each containing six membrane-spanning domains (S1–S6). Carboxyl and amino termini are present in the cytoplasmic region. The pore region is located between S5 and S6 and is highly selective to potassium ions, and the S4 domain acts as a voltage sensor [19,20]. The N-terminus includes a calmodulin (CaM) binding site and a Per-Arnt-Sim (PAS) domain, which has been associated in other proteins with oxygen sensing and the activation of hypoxia inducible factor (HIF1) [3,21–23]. The C-terminus includes a cyclic-nucleotide binding domain (cNBD), a tetramerization-coil-coil domain, and binding sites for calmodulin (CaM) and calcium/CaM-dependent protein kinase II (CaMKII) [24–27]. Interestingly, a nuclear localization sequence (NLS) has been found in Eag1 channels and currents resembling Eag1 channel activity blocked by astemizole have been recorded in the inner nuclear membrane [28]. One of the most attractive features of Eag1 channels is their oncogenic properties, which has raised many investigations in cancer research and led to the proposal that these channels are cancer markers.

## 3. Eag1 Oncogenic Potential

The first indications that Eag1 might have a role in the cell cycle came from observations in *Xenopus* oocytes, which showed that the electrophysiological properties of the channel change when the cell cycle progresses. In such a model, Eag1 channel amplitude was reduced when oocyte maturation was induced by either progesterone or mitosis promoting factor (MPF) [29]. Subsequently, it was suggested that Eag1 channel regulation during the M phase might be associated with cytoskeleton re-arrangements [30]. The oncogenic potential of Eag1 was discovered when the transfection of Eag1 into cells that normally do not express the channel induced a transformed phenotype [16]. Eag1-transfected cells were able to grow in foci and in the presence of low serum concentrations. In addition, Eag1-transfected cells lost cell-contact inhibition and produced friable tumors when injected into immunosuppressed mice [16]. In such seminal work, Eag1 mRNA was found to be expressed in cancer cell lines from neuroblastoma, breast, and cervical cancers. A major question was whether Eag1 channel expression in cancer cell lines was associated with a potential role for the channel in cell proliferation or if such expression was only a consequence of the malignant phenotype. Inhibition of channel expression with antisense oligonucleotides led to a reduction in DNA synthesis in cancer cells, supporting the oncogenic potential of Eag1 channels [16]. Since then, several approaches inhibiting either channel expression or activity by siRNA, drugs (astemizole, imipramine) and monoclonal antibodies has led to inhibition of proliferation of tumor cells both *in vitro* and *in vivo* [18,31–34]. It has been shown that Eag1 interferes with hypoxia homeostasis and induces angiogenesis in tumors [34]. Although the precise mechanistic link between Eag1 and cell proliferation remains elusive, the oncogenic potential of Eag1 channels has made these proteins very interesting cancer targets.

## 4. Eag1 as a Cancer Marker

Eag1 expression has been found in many cancer cell lines from different tissue types, including cells from neuroblastoma, breast, melanoma, colon, lung, cervical, and ovarian cancers [16,18,35–37]. Because of the restricted distribution of Eag1 in normal tissues, it was very important to know if the channel could be detected not only in cell lines but in human tumor biopsies. Overexpression of Eag1 channels in many tumor biopsies has emphasized the potential use of this channel as a cancer marker. In contrast to the corresponding normal tissues, Eag1 has been reported to be expressed in hundreds of human biopsies from different malignancies, including cervical, breast, lung, liver, prostate, colon, ovarian and gastric cancers; gliomas; leukemia and different types of sarcoma [17,36–43]. Gliomas represent an interesting case because high expression of Eag1 was found in low-grade gliomas, whereas low Eag1 expression was observed in malignant gliomas. This may be due to the typical low cellular differentiation of the latter [39]. It is worth mentioning that in a cervical cancer study [38], one of the biopsies obtained as a normal “control” tissue was from a patient subjected to a hysterectomy because of a disorder not associated with any type of tumor and who had a negative Pap smear. However, strong Eag1 expression was found in this patient. Post-surgical histopathological studies revealed a cervical carcinoma, which had not been detected with the traditional screening method. Despite this was only one case in such study, it illustrates the clinical relevance that Eag1 channels might have as cancer markers. Cervical cancer is a very good example of how Eag1 detection might be included in cancer screening programs because cervical sample collection is a standard method for cancer screening. Eag1 detection in other tissues that are not easily accessible might be overcome by using labeled antibodies and different imaging studies. This approach has been used *in vivo* to detect non-palpable tumors in mice [5]. The restricted distribution of Eag1 channels in normal human tissues and the more abundant and ubiquitous expression in human tumor biopsies provide a promising tool for cancer diagnosis based on the detection of Eag1.

## 5. Eag1 as a Potential Early Tumor Marker

Molecular and clinical studies have shown that Eag1 channels might also be used as early biomarkers. An “early” cancer marker should be detected in either pre-malignant lesions or in conditions potentially leading to cancer. In addition, such an early marker is expected to be regulated by cancer etiological factors. Eag1 channels seem to fulfill these requirements. The first suggestion of Eag1 as a potential early tumor marker was from studies in cervical biopsies [38]. Eag1 mRNA was detected in cervical biopsies from patients with normal pap smears. However, one of them had human papilloma-virus (HPV) infection, which is the main etiological factor for cervical cancer. Another patient had an ovarian tumor, and another had hyperplasia in the endometrium. Eag1 expression under these conditions led the researchers to suggest Eag1 channels as potential early tumor markers [38]. Later, *in vitro* studies demonstrated that HPV oncogenes might regulate Eag1 expression. Normal keratinocytes lacking HPV oncogenes do not express Eag1; however, keratinocytes forced to express the HPV oncogenes E6 and E7 displayed strong Eag1 mRNA and protein expression [18,43]. HPV infection is proposed to be necessary but not sufficient to induce cervical cancer, and other factors have been suggested to be involved, especially estrogens. Interestingly, estrogens also up-regulate Eag1 expression. This regulation seems to depend on the presence of the estrogen receptor-α because cervical cancer cells lacking this receptor did not display estrogenic regulation [18]. Detection of Eag1 channels has also been reported in pre-malignant cervical lesions. Channel expression was found in 67% of the cervical cytologies from low-grade intraepithelial lesions and in 92% of the samples from high-grade intraepithelial lesions but only in 27% of the normal samples. Notably, morphologically normal cells obtained from dysplastic samples also exhibited Eag1 expression [43]. This is important because in some cases, only morphologically normal cells are collected despite the presence of an intraepithelial lesion. Consequently, the cytopathologist describes the sample as normal. Eag1 expression might serve as an indicator to recommend a closer follow-up of the patient. The observation that Eag1 channel expression is regulated by estrogens led to the study of Eag1 expression in cervical cytologies from patients using estrogens. Interestingly, almost 50% of the normal patients taking estrogens displayed Eag1 expression, while only 20% of the patients not taking estrogens displayed cervical Eag1 expression [43]. All of these findings strongly suggest Eag1 as an early biomarker of cervical dysplasia. Because estrogen use has been considered a potential risk factor for developing cervical cancer, Eag1 detection in patients using estrogens might be an indicator suggesting that these patients might be at risk of developing cervical lesions [43]. In addition, the regulation of Eag1 by HPV oncogenes suggests that Eag1 might be an early marker in other types of tumors affected by HPV, including lung, head, and neck cancer.

Eag1 might also be an early marker for breast and colon cancer. Eag1 expression was found in the surrounding “tumor-free” tissue from breast cancer biopsies, in contrast with the absence of Eag1 mRNA expression in normal tissue [17]. Eag1 expression was also found in biopsies from diverticulitis, which has the potential to develop into colon cancer [40]. Finally, Eag1 was found to be overexpressed in a mouse model of colon cancer following exposure to chemical carcinogens [40]. Probably Eag1 has a major general role responding to potential cell damage, which in many cases leads to inflammation and cancer. Actually, cancer has been strongly associated to inflammation in several tissues. In summary, Eag1 can be detected in premalignant lesions, and Eag1 is regulated by cancer etiological factors, including HPV oncogenes, hormones and chemical carcinogens, making Eag1 a potential early marker for different types of cancer.

## 6. Eag1 as a Prognosis Marker

Prediction of either a cancer patient′s survival or response to anti-cancer therapy is a major challenge in oncology. Several studies suggest Eag1 as a prognostic marker. Colon cancer patients displaying Eag1 amplification had a poor survival [40], compared to patients with no Eag1 amplification. A similar observation has been found in acute myeloid leukemia in which channel expression strongly correlated with increasing age, higher relapse rates and significantly shorter survival [42]. Finally, a study on ovarian cancer patients showed that high expression of Eag1 is significantly associated with poor survival, tumor grade and the presence of residual disease [36]. The molecular mechanism of how Eag1 amplification/overexpression is associated with poor survival remains unknown; nevertheless, Eag1 might potentially serve as a prognostic marker for at least some types of cancer. Validation of this association would be very interesting to be done for several types of cancers.

## 7. Clinical Implications

The restricted distribution of Eag1 channels in normal tissues, the more abundant and ubiquitous expression in tumors, and the oncogenic properties of the channel make Eag1 a potential tool for the detection of different types of cancer. The presence of Eag1 in pre-malignant lesions or in tissues potentially leading to cancer, as well as the regulation of Eag1 by cancer etiological factors, cause this channel to be a potential early marker for several types of tumors. Table 1 summarizes examples of the potential use of Eag1 as a biomarker in oncology. A major problem in cancer is the detection of tumors at curable stages. Monoclonal antibodies have been shown to detect Eag1 in a very specific manner [33]; thus, Eag1 might be an important tool in detecting cancer. It has been shown that Eag1 expression is regulated by the p53/miR34/E2F pathway [44]. Because cells transfected with the E6-HPV oncogene shows high Eag1 expression [18] and because E6 inactivates p53, the mechanistic link between Eag1 expression and HPV might be the inactivation of p53 by E6. The elucidation of the mechanistic link between Eag1 expression and other cancer etiological factors should help to emphasize the use of Eag1 as an early tumor marker in other tissues. Eag1 expression mainly in cancer cells can be used to direct anti-cancer therapy not only by directly targeting Eag1 as described above but also to direct other therapies to cancer cells. Recently, a strategy based on an Eag1 antibody was designed to produce apoptosis in cells expressing Eag1 [45]. It will also be important to know if Eag1 expression can be inhibited as a potential chemopreventive approach. For instance, calcitriol, the active metabolite of vitamin D with known antiproliferative effects, down-regulates Eag1 expression in breast tumor-derived cells and in cervical cancer [46,47].

## 8. Conclusions

Despite the hundreds of clinical trials that are currently being conducted for cancer patients, most new anticancer drugs fail to pass Phase I studies. New early tumor markers are needed to treat the disease at curable stages. In addition, new therapeutic targets are required to treat patients who are not responding to available treatments. Despite further mechanistic, exploratory and validation studies are necessary, Eag1 currently is considered as a promising early tumor marker, cancer marker and prognostic marker.

## Figures and Tables

**Table 1.** Eag1 channels as potential biomarkers in oncology.

**Cancer Type**	**Early Biomarker**	**Prognostic Marker**	**Tumor Marker**	**References**
Cervical cancer	*		*	[37,42]
Gliomas			*	[38]
Gastric cancer		*	*	[36]
Sarcomas			*	[40]
Ovarian cancer		*	*	[35]
Colon cancer	*	*	*	[39]
Acute myeloid leukemia		*	*	[41]
Breast cancer	*		*	[17]
Lung cancer			*	[17]
Prostate cancer			*	[17]
